# Therapeutic Dose of Hydroxyurea-Induced Synaptic Abnormalities on the Mouse Spermatocyte

**DOI:** 10.3389/fphys.2021.666339

**Published:** 2021-07-09

**Authors:** Xiaobo Fan, Yunxia Zhu, Naixin Wang, Bing Zhang, Cui Zhang, Yanan Wang

**Affiliations:** ^1^Laboratory of Molecular Cytogenetics, School of Bioengineering, Xuzhou University of Technology, Xuzhou, China; ^2^The Center of Reproductive Medicine, Xuzhou Maternity and Child Health Care Hospital, Xuzhou, China

**Keywords:** hydroxyurea, sickle cell disease, meiosis, synaptonemal complex discontinuities, unrepaired double strand breaks

## Abstract

Hydroxyurea (HU) is a widely used pharmacological therapy for sickle cell disease (SCD). However, replication stress caused by HU has been shown to inhibit premeiotic S-phase DNA, leading to reproductive toxicity in germ cells. In this study, we administered the therapeutic doses of HU (i.e., 25 and 50 mg/kg) to male mice to explore whether replication stress by HU affects pachytene spermatocytes and causes the abnormalities of homologous chromosomes pairing and recombination during prophase I of meiosis. In comparison with the control group, the proportions of spermatocyte gaps were significantly different in the experimental groups injected with 25 mg/kg (*p* < 0.05) and 50 mg/kg of HU (*p* < 0.05). Moreover, the proportions of unrepaired double-stranded breaks (DSBs) observed by γH2AX staining also corresponded to a higher HU dose with a greater number of breaks. Additionally, a reduction in the counts of recombination foci on the autosomal SCs was observed in the pachytene spermatocytes. Our results reveal that HU has some effects on synaptonemal complex (SC) formation and DSB repair which suggest possible problems in fertility. Therefore, this study provides new evidence of the mechanisms underlying HU reproductive toxicity.

## Introduction

Sickle cell disease (SCD) affects approximately 4.4 million people worldwide and is most prevalent in Asia and Africa. SCD is an inherited blood disorder on chromosome 11 caused by a β-globin gene mutation from GAG to GTG that results in an amino-acid substitution (i.e., glutamic to valine), which forms the abnormal sickle hemoglobin (HbS). HbS alters the erythrocyte membrane causing vaso-occlusion and disrupting endothelial cell function. This situation places patients with SCD at increased risk of episodic ischemia, resulting in hypoxia to vital organs such as the brain, skeleton, and liver. Another symptom of SCD is chronic and episodic pain in patients. SCD cannot be cured, but hydroxyurea (HU) is used as a chemical chemotherapy compound to control this disease. Since the middle of the 1980s, HU has been used as a clinically effective pharmacological therapy to treat adult patients with SCD. In the year 2002, HU was suggested for the treatment of children with SCD. Treatment with HU generally results in the increased fetal hemoglobin (HbF) level, the decreased leukocyte count, and the expression of cell adhesion proteins which regulate pain and disease severity (Lanzkron et al., 2008).

The effect of HU-stimulated HbF *in vivo* remains unclear. It has been shown that HU impedes S-phase DNA synthesis and repair, due to the reduction of ribonucleotides to deoxyribonucleotides (McGann and Ware, 2015). This causes hematopoietic arrest and altered erythroid kinetics from the recruitment of erythroid progenitors to maintain a relatively high level of HbF (Wyrobek and Bruce, 1975; Arlt et al., 2018). Although adverse effects remain a concern, the short-term effects of HU are minimal and only cause reversible toxicities on growth and development. Even in the long-term study of HU treatment, patients with SCD did not show serious complications. Thus, HU therapy is considered relatively safe and effective for both children and adults with SCD (Lanzkron et al., 2008; Strouse and Heeney, 2012).

In spite of this, there is concern that HU treatment has a detrimental effect on the fertility of patients with SCD. Some studies have examined if HU has reproductive toxicity in men or male mice. Lanzkron et al. (2008) reported that HU treatment in mice caused decreased testis weight and sperm count and proposed a possibility of spermatogenesis disruption in male patients with SCD treated with the therapeutic doses of HU. In adult male mice, HU treatment caused testicular damage and increased abnormal sperm morphology (Wyrobek and Bruce, 1975; Arlt et al., 2018). Subsequently, the study of male patients with SCD treated with HU showed abnormal sperm parameters and a possible increase in the incidence of oligospermia (Jones et al., 2009). In addition, HU-stalled replication forks that collapse into double-stranded breaks (DSBs) have the potential to form copy number variants (CNV) in mitotic cells (Petermann et al., 2010; Arlt et al., 2011). Arlt et al. (2011, 2018) administered HU to mice by oral gavage and subcutaneous pump. They found that HU treatment caused tissue and genetic toxicity but did not increase CNV formation during mammalian spermatogenesis by tissue histopathology and reticulocyte micronucleus assays (Arlt et al., 2011, 2018). It is understood that DNA only replicates in S-phase spermatogonia and preleptotene spermatocytes during spermatogenesis (Wakayama et al., 2015) and that only cells under the low levels of replication stress can progress through the cell cycle (Culligan et al., 2004). In yeast, HU effectively inhibited premeiotic S-phase DNA and zygotene synthesis arrests, resulting in decreased meiotic recombination (Barbera and Petes, 2006; Kugou et al., 2009). Therefore, replication stress by HU might impact on the chromatin structure of preleptotene spermatocytes (Jones et al., 2009; Arlt et al., 2018). However, how HU affects the meiotic process is still unclear. At present, there was no clear evidence to suggest that HU disrupts spermatogenesis, especially in prophase I meiotic events. In this study, we treated male mice with a clinically relevant dose of HU to evaluate the hypothesis that mice spermatocytes treated with HU may impact on homologous chromosomes pairing and recombination during prophase I of meiosis causing abnormalities with fertility.

## Materials and Methods

### Animals and Sample Collection

Fifteen male ICR mice (i.e., 4 weeks old) were purchased from Beijing Vital River Laboratories (Beijing, China). Under normal light–dark cycles, mice were housed at 24–26°C and given *ad libitum* access to food and water. After HU injection, the physical state of the mice was monitored every 4 h. Pain relief was administered if clinical or toxicological symptoms appeared. If abnormal breathing, head tilt, lethargy, or paresis occurred, then euthanasia was carried out by cervical dislocation. In this study, the procedures of animal care and sample collection were maintained in accordance with the guidelines of the Laboratory Animal Center, Xuzhou University, Xuzhou, China. The use of animal in our study was reviewed and approved by the Animal Care Committee at the University of Xuzhou (approval No. 20191202-019).

### Hydroxyurea Administration by Intraperitoneal Injection

Four-week-old male mice were injected intraperitoneally with a clinically relevant dose of HU two times daily for a period of 14 days. The HU was dissolved in phosphate-buffered saline (PBS), and a fresh solution was prepared before each experiment at a final concentration of 25 and 50 mg/kg. Mice were randomly divided into three groups with five mice each. Mice in the two experimental groups were treated with intraperitoneal (IP) injection of 25 mg/kg of HU as the low dose and 50 mg/kg of HU as the high dose every day, respectively. Mice in the control group were injected with the equivalent volume of PBS.

### Histological Analysis of Testicular Tissue

The mice were killed by cervical dislocation and weighed after 14 days of injection. One testis was weighed and prepared for the spreads of spermatocyte nuclei, and the other was fixed in 4% paraformaldehyde at 4°C for paraffin sections. Then, the tissues were stained with H&E. Images of the sectioned slides were captured by the light microscopy (Z51; ZEISS, Germany) with 20 × magnification. The testicular tissue measurements were obtained from seminiferous tubules. The testicular architecture (i.e., the proportion of seminiferous tubules) was evaluated in accordance with the published testicular development protocols in the mouse (Montoto et al., 2012). The changes in the morphology of the testicular tissue in mice treated with HU were analyzed statistically.

### Chromosome Spreads

Spermatocyte nuclei were spread by a modified method (Dia et al., 2017). Seminiferous tubules were placed into cold hypotonic extraction buffer (i.e., 30 mM Tris–HCl at pH 8.2, 50 mM sucrose, 17 mM trisodium citrate dihydrate, 5 mM ethylenediaminetetraacetic acid, 2.5 mM dithiothreitol, and 1 mM phenylmethanesulfonyl fluoride) and incubated on ice for 30–45 min. After incubation, tubules were moved into a watch glass containing 100 mM of sucrose solution and minced to obtain the cloudy solution. Before chromosome spread, slides were soaked in fixative solution (i.e., 4% paraformaldehyde at pH 7.2) at 4°C for 12 h. The cell suspension was mixed with a fixative droplet on the slide corner. Then, the slide was tilted to spread the mixture across the whole slide surface. The spread slides were placed in a humidified chamber to dry at room temperature for 2 h. After drying, slides were washed with 0.4% Photo-Flo 200/1X PBS and rinsed with distilled water. The washed slides were transferred to a dark environment to dry for 20 min at room temperature for immunofluorescence staining.

### Immunofluorescence Staining of Chromosome Spreads

Immunofluorescence staining was performed as described in the previous studies (Yoon et al., 2018). The primary antibodies used in this study included as follows: mouse anti-SCP3 (1:600, ab97672, Abcam), rabbit anti-SCP1 (1:600, ab15090, Abcam), rabbit anti-γH2AX (1:300, ab26350, Abcam), and rabbit anti-MLH1 (1:200 ab92312, Abcam). The secondary antibodies used were goat anti-rabbit Alexa 488/594 (1:300, ab150077/ab150080, Abcam) and goat anti-mouse Alexa 488/594 (1:300, ab150113/ab150116, Abcam). Nuclear DNA was stained by 4′,6-diamino-2-phenylindole (DAPI). Images were captured by the fluorescence microscopy Imager M2 (ZEISS, Gottingen, Germany) at 100 × oil objective magnification and an Axiocam 503 monochrome camera.

### Chromosome Spread Analysis

Images of SYCP3/SYCP1/γH2AX/MLH1 signals on synaptonemal complex (SC) were measured by ImageJ 1.8.0 software^1^. The percentage of SC length as a relative position represented the distance from the centromere to gaps/γH2AX/MLH1 foci on SC. According to the morphology of the XY pair, the identification of synapsis and DSBs in the pachytene nuclei was classified as early or late depending on its occurrence. The heterochromatin around the centromeres was indicated by DAPI staining. The absolute/average relative SC lengths for the experimental and control groups were measured per cell and classified into five groups based on similar SC lengths as follows: SCs 1–2, 3–5, 6–11, 12–16, and 17–19 (Supplementary Table 1). This criterion is similar to the classification of a continuous gradation in relative SC lengths (Anderson et al., 1999). MLH1 foci were considered to locate on pachytene SCs when the overlap between two fluorescent signals appeared, but not on zygotene or diplotene SCs, as previously described (Anderson et al., 1999).

### Statistical Analysis

The data on relative SC lengths, area of seminiferous tubule lumen, testis weight, and MLH1 foci in mice injected with 25 and 50 mg/kg of HU were compared with controls by using the Mann–Whitney *U*-test. The data collected in immunofluorescence staining of chromosome spreads (SCP1/SCP3/γH2AX) were analyzed by using the Fisher’s exact test.

## Results

### Histological Analysis of Testis After HU Treatment

**FIGURE 1:**
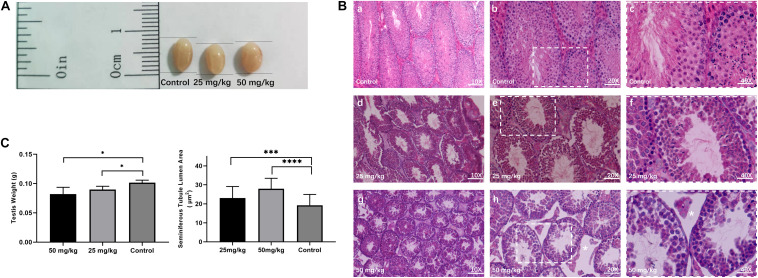
Testicular histomorphology of mice after HU treatment. **(A)** Representative image of testes from control and mice injected with 25 and 50 mg/kg of HU. **(B)** The H&E staining of the testis from control and HU-injected mice. Normal mice spermatogenesis present in control testes (a, b, and c, magnification bar = 10×, 20×, and 40×, respectively). Decreased germinal epithelium area was observed in mice injected with either 25 mg/kg (d, e, and f) or 50 mg/kg (g, h, and i) of HU. c, f, and i show that the rectangular area with the white dotted lines in b, e, and h delineates the higher magnification image. As previously described, white stars indicate that loss of fluid in the seminiferous tubules due to decreased testosterone in mice injected with 50 mg/kg of HU (Jones et al., 2009). Bars a, d, and g: 100 μm; d, e, and h: 50 μm; and c, f, and i: 25 μm. **(C)** Mean testis weight and area of seminiferous tubule lumen from controls and mice injected with 25 and 50 mg/kg of HU. Five mice were analyzed for each group, and the data as mean ± SD were compared between groups using the Mann–Whitney *U*-test. Asterisks denote significance (^∗∗∗^*p* < 0.0005, ^****^p < 0.0001 and ^∗^*p* < 0.05).

To investigate the testicular histopathology of mice treated with the low dose of HU (i.e., 25 mg/kg) and the high dose of HU (i.e., 50 mg/kg), the histological analysis of testicular tissue from experimental and control groups was performed (Figure 1). HU-treated mice showed degenerative changes in the testes. Compared with the control group, an increase in the area of seminiferous tubule lumen was observed in mice treated with 25 mg/kg of HU (*p* < 0.001) and 50 mg/kg of HU (*p* < 0.0001). In the high-dose group, there were decreased numbers of sperm and germ cells. In the low-dose group, the testes had a slightly decreased amount of mature spermatids, and the tubules had slight changes in the number of germ cells, suggesting that not all seminiferous tubules were affected. Compared with the high dose, there was a smaller spacing in seminiferous tubules in the low dose. Additionally, the experimental groups (i.e., 25 and 50 mg/kg of HU) also showed a reduction (*p* < 0.01) in testis weight compared with the controls.

### Gap Formation in Autosomal Pachytene SCs After HU Injection

**FIGURE 2:**
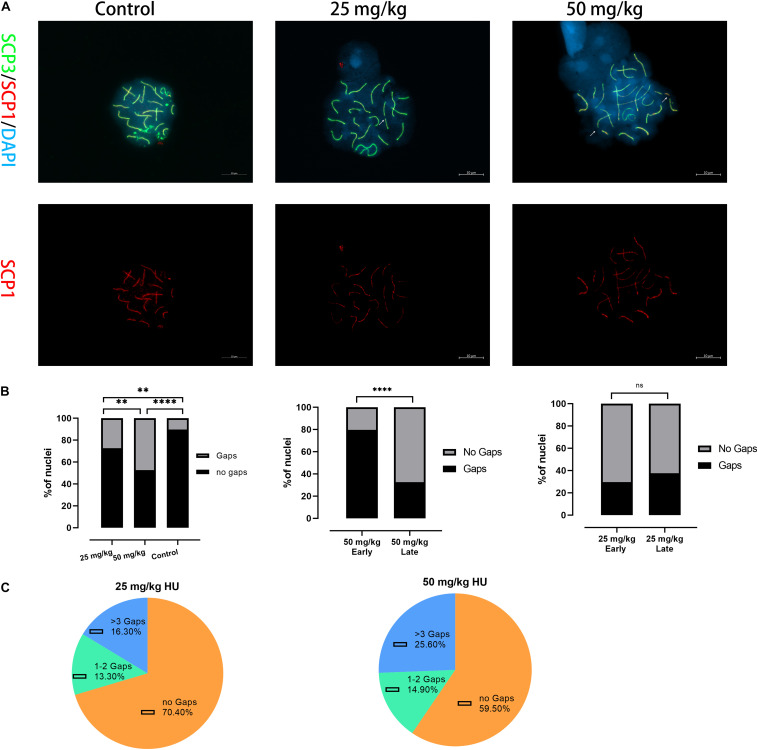
Alterations in the synapsis process in HU-exposed male mice. **(A)** Pachytene spermatocyte chromosome spreads were immunostained with antibody SCP3 (green) and SCP1 (red) in controls and mice injected with 25 and 50 mg/kg of HU. Of note, mice injected with 25 and 50 mg/kg of HU show synaptic abnormalities. Arrows indicate example gaps (i.e., SC discontinuities). Scale bars = 10 μm. **(B)** Quantification of the proportions of SC gaps in pachytene nuclei after HU injection; mice injected with 29.5% of 25 mg/kg and 40.4% of 50 mg/kg of HU, compared with 11.5% of controls pachytene nuclei (*n* = 100; using the Fisher’s exact test, ^∗∗^*p* < 0.01, ^****^p < 0.0001) show spermatocytes with gaps. ns indicates no significant difference between the two doses of HU injection (*p* > 0.05). **(C)** The percentage of gaps (1–2 gaps, more than 3 gaps, and no gaps) on SCs in mice injected with 25 and 50 mg/kg of HU.

Then, we tested whether spermatocytes of mice treated with HU could cause synaptic anomalies. We observed that autosomal SC presented gaps in the pachytene cells (Figure 2A). Mice treated with the high dose presented a higher percentage of SCs with gaps than the low dose (*p* < 0.01) and controls (*p* < 0.0001). Moreover, the number of gaps of all autosomal SCs was analyzed in the early and late pachytene stages. In the high-dose group, the gaps were predominantly presented in the early pachytene stage rather than the late pachytene stage (Figure 2B). However, there was no significant difference in the proportion of gap presentation between the early and late pachytene stages in the low dose. In the low dose, 29.6% of cells had synaptic anomalies with 13.3% of these nuclei showing 1–2 gaps and 16.3% showing three or more gaps (Figure 2C). In the high-dose group, the number of gaps increased up to 40.4%, with 14.9% of nuclei showing 1–2 gaps and 25.6% showing three or more gaps (Figure 2C). Additionally, we observed that the number of gaps was not only related to the dose of HU but also related to SC length. The five long-length SCs (i.e., 1–5) presented a higher number of gaps than the mid-length SCs 6–11.

The cumulative distribution curves of gaps for experimental groups are presented (Supplementary Figures 1A, 3). The orientation of graphs is from the centromere on the left to the telomere on the right. We found that the distributions of gaps along SCs showed no significant difference between high- and low-dose groups (*p* > 0.05, using the Kolmogorov–Smirnov test). In the high dose, the distributions of gaps for the long-length SC groups (SCs 1–2 and 3–5) peaked closest to the center of the SCs. For mid-length SCs 6–11, the distribution of gaps appeared to be bimodal. For short-length SCs 12–16 and 17–19, one distribution peak is located in the 30% interval to the centromere. Compared the distributions of gaps of each SC group with the distributions of every other group, there was no significant difference among SC groups in the high dose (*p* > 0.05, using the Kolmogorov–Smirnov test). In the low dose, gaps along the long-length SCs 1–2 and 3–5 were distributed near the centromere. For SCs 6–11, the distribution of gaps was bimodal. For short-length SCs 12–16 and 17–19, one distribution peak is located in the center of the SC length. There was no significant difference among SC groups in the low dose (*p* > 0.05, using the Kolmogorov–Smirnov test).

### The Accumulation of Unrepaired DSB on the Autosomes

**FIGURE 3:**
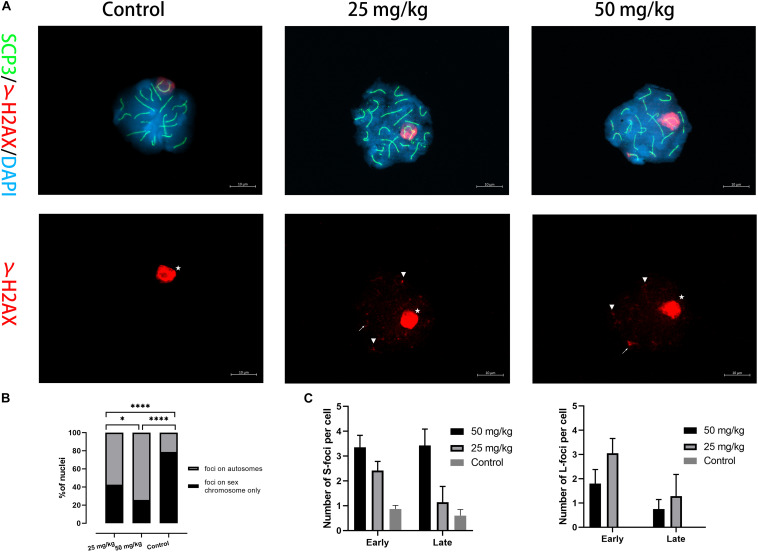
The presence of γH2AX foci on autosomal SCs. **(A)** Immunostaining for γH2AX (red) and SCP3 (green) in mice injected with 25 and 50 mg/kg of HU stained in pachytene spermatocyte chromosome spreads. A single-channel TexRed image for γH2AX is also presented. Asterisks indicate sex bodies. An arrow indicates L-foci, and arrowheads indicate S-foci associated with axes. Scale bars = 10 μm. **(B)** Quantification of the proportions of γH2AX foci in autosome and sex chromosomes of pachytene nuclei after HU injection; mice injected with 59.2% of 25 mg/kg and 75.7% of 50 mg/kg of HU, compared with 23.1% of controls pachytene nuclei (*n* = 70; using the Fisher’s exact test, ^****^p < 0.0001) obtained from five mice of each group showed spermatocytes with γH2AX foci. **(C)** Quantification of γH2AX foci containing S-foci and L-foci in early and late pachytene cells of mice injected with 25 and 50 mg/kg of HU (i.e., black and gray bars, respectively) showed spermatocytes with γH2AX foci. Bars represent mean ± SEM. The statistical analysis between mice injected with 25 and 50 mg/kg of HU at early and late pachytene (*n* = 70) was performed using the Mann–Whitney *U*-test, and ns indicates no significant difference between the two doses of HU injection (*p* > 0.05). *p < 0.05.

We then examined whether the DNA damage caused by unrepaired DSB occurred in pachytene spermatocytes after HU treatment. γH2AX was used as a marker of DNA damage located within unsynapsed regions and DSB regions (Figure 3A). γH2AX signals on autosomal synapsis were grouped into two types, namely, small γH2AX foci (S-foci) and larger γH2AX foci (L-foci) as recently described (Crichton et al., 2018). In this study, γH2AX signals were found in unsynapsed sex chromosomes and autosomal SCs in pachytene nuclei (Figure 3A). The results showed that the low dose (59.2%, *n* = 70; *p* < 0.05) and the high dose (75.7%, *n* = 70; *p* < 0.05) had greater percentages of pachytene nuclei with γH2AX foci of autosomal SCs compared with controls (Figure 3B). Moreover, we observed that the number of γH2AX foci on autosomal SCs in the high dose was significantly different from that of the low dose (*n* = 70; *p* < 0.05), suggesting that the number of γH2AX foci was related to the dose of HU treatment (Figure 3B). Furthermore, the number of γH2AX signals on each SC group was analyzed and showed that the higher number of γH2AX signals on SCs 6–11 presented 32 foci in the low-dose group and 55 foci in the high-dose group. In addition, we compared the low- and high-dose groups for their S-/L-foci at the pachytene stage. We found a greater number of S-foci of the early and late pachytene spermatocytes in the high dose (i.e., S-foci: 3.35 ± 2.94 per SC) compared with the low dose (i.e., S-foci: 2.42 ± 2.05 per SC). Moreover, in the low dose, both S-foci and L-foci had a higher number at early pachytene than at late pachytene. By contrast, in the high dose, there were more L-foci at early pachytene than at late pachytene (Figure 3C). The cumulative distribution lines of γH2AX foci on autosomal SCs are displayed according to their relative SC length (Supplementary Figures 1B, 3). The distributions of γH2AX signal along autosomal SCs showed no significant difference between high- and low-dose groups (*p* > 0.05, using the Kolmogorov–Smirnov test). For all SCs, there was a common distribution peak of γH2AX signals that appeared in the 10% interval near the telomere. Moreover, a distribution peak of γH2AX signals in the long- and mid-length SC groups appeared near the centromere. For the short-length SCs 17–19, γH2AX signals were distributed near the telomere.

### Analysis of MLH1 Foci on Nuclei

**FIGURE 4:**
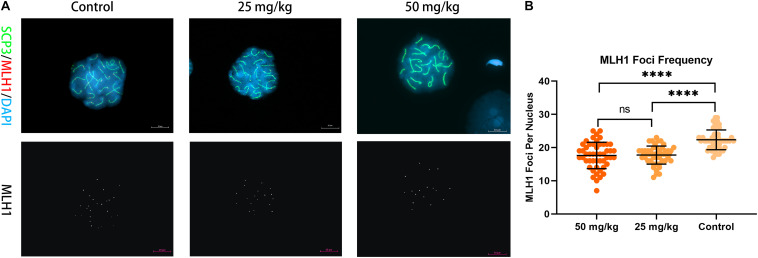
Recombination foci in pachytene spermatocytes in HU-exposed male mice. **(A)** Pachytene spermatocyte chromosome spreads were immunostained with antibody SCP3 (green) and MLH1 (red) in mice injected with 25 and 50 mg/kg of HU and controls. Mice injected with 25 and 50 mg/kg of HU show a reduction in recombination foci. MLH1 foci are shown in white on the lower panel of **(A)**. Scale bars = 10 μm. **(B)** Quantification of the MLH1 foci per pachytene nuclei; MLH1 foci frequency from control and mice injected with 25 and 50 mg/kg of HU. Five mice were analyzed for each group, and the data as mean ± SD were compared between groups using the Mann–Whitney *U*-test. Asterisks denote significance (*n* = 100; ^****^p < 0.0001).

Then, to analyze whether unrepaired DSBs cause changes in the number and frequency of recombination foci in pachytene spermatocytes after HU injection, we stained chromosome spreads from HU-injected mice groups and controls using antibodies to MLH1 (Figure 4). MLH1 foci as indicators of meiotic recombination progress to mark crossover (CO) sites are scored in autosomal SCs and XY pairs (Crichton et al., 2018). There were 22 ± 3 MLH1 foci in control pachytene autosomal spermatocytes. Interestingly, the average number of MLH1 foci in the high dose (i.e., 17 ± 4 foci) and the low dose (i.e., 17 ± 3 foci) of HU-injected groups was significantly different from controls (*p* < 0.0001, using the Mann–Whitney *U*-test). The distribution of MLH1 foci was mapped on the pachytene nuclei. The relative SC length (%) also represents the position of MLH1 foci (Supplementary Figures 2, 3). The distributions of MLH1 foci along SCs showed no significant difference between high- and low-dose groups (*p* > 0.05, using the Kolmogorov–Smirnov test). In the high- and low-dose groups, the distribution peaks appeared near the center and the distal end of SCs 1–2. The bimodal distributions appeared on SC groups 3–5, 6–11, and 12–16, one distribution peak near 30% of SC lengths, and the other peak near telomere. Additionally, for the short-length SCs 17–19, there was one distribution peak near the sub-telomeric region.

## Discussion

### Synaptic Abnormalities in Mice Treated With HU

The histological analysis of testicular tissue from experimental and control groups showed the degenerative changes in the mice testes treated with HU, such as an increase in the area of seminiferous tubule lumen and a decrease in the numbers of sperm and germ cells. These observations are consistent with the previous report that pre-meiotic spermatogonia and stem cells appeared to be highly sensitive to HU, and decreased spermatogenesis manifested as severe depletion of round and elongating spermatids, especially moderate to marked decreases in pachytene spermatocytes (Arlt et al., 2018).

The three-dimensional structure of SC consists of lateral elements (formed by SCP3) and transverse filaments (formed by SCP1), with the transverse filaments separating the lateral elements. During the pachytene stage of meiosis, the SC is presented between homologous chromosomes and is disassembled during the diplotene stage (Qiao et al., 2012). Our results show that HU treatment causes gap formation on autosomal SCs. This failure of SC formation is accompanied by a discontinuous loading of SC proteins, which shows a more fractured appearance in SCP1 or SCP3 mutant spermatocytes (Murdoch et al., 2013; Dunce et al., 2018), indicating HU treatment could affect the efficient loading of SC proteins that are necessary for continued synapsis. In this study, male mice were treated with two clinically relevant doses of HU. The immune-cytogenetic analysis of mice synapsis has been carried out by SCP1 and SCP3 immunofluorescent staining. If pachytene nuclei exhibited SC gaps, the cell was recorded as a synaptic defect (Murdoch et al., 2013). We found that autosomal pachytene SCs of mice in the high-dose group (40.4%) had more gaps than in the low-dose group (29.5%) and controls (11.5%). These results indicated that the high-dose HU-treated mice presented a greater number of gaps. The increased gap in HU-treated cells is likely to reflect synaptic abnormalities associated with altered gene expression (Bolcun-Filas et al., 2009), causing discontinuous loading of SC proteins. Notably, gaps have also been reported in subfertile males (Vidal et al., 1982). Men with infertility had discontinuities (i.e., gaps) and unpaired regions (i.e., splits) at a higher rate than normal men due to synaptic abnormalities at the pachytene stage (Codina-Pascual et al., 2006). Furthermore, we found that the number of gaps is related not only to the dose of HU but also to the SC length. A greater number of gaps are predominantly located in SCs 1–5. A possible explanation is that the greater number of gaps in the long-length SC groups is probably related to their bigger size. These mice SCs homologous with human metacentric and acrocentric chromosomes have been previously noted to be frequently affected by gaps (Sun et al., 2005). In addition, this study revealed that most gaps appeared in the early pachytene stage. However, Voelkel-Meiman et al. (2013) reported that a discontinuous SC assembly was observed in the late pachytene phase. It is a possible explanation that HU-stalled replication forks inhibited premeiotic S-phase DNA and caused zygotene synthesis arrests, leading to the elevated degrees of chromosome loss in the early pachytene stage (Kugou et al., 2009). Additionally, discontinuous regions (i.e., gaps) could cause transcriptional silence in the pachytene phase relative to synapsed regions where the spermatocytes are treated by chemical agents (Sun et al., 2005). These synaptic anomalies are particularly prone to breaks resulting in sperm chromosome aberrations and DNA fragmentation (Wong et al., 2008). We proposed that HU treatment could induce discontinuous SC (i.e., gaps) formation in autosomal SCs during the pachytene stage.

The distribution of gaps on autosomal SCs was also analyzed. SC gaps induced by two doses presented similar distribution patterns. A greater number of gaps are located in the 30% interval region to the centromere in the high-dose group, whereas the distribution of gaps in the low-dose group mainly appeared near the 40% interval of SCs. Thus, the number of gaps tended to move toward the non-centromeric region along the SC with the increasing dose of HU. Our data confirmed the previous results that SC gaps were distributed more frequently within the non-centromeric regions. These regions were related to the severe abnormalities of the synaptic process (Dunne and Davies, 2019). In addition, we examined whether the failure of SC formation is accompanied by unrepaired DSB.

### γH2AX Foci on Autosomal SCs

Hydroxyurea inhibits ribonucleotide reductase causing DSBs and stalled fork collapses, leading to chromosomal aberrations such as gaps, breaks, and polyploidy (Xu et al., 2011; Covo et al., 2012). As a marker for DSBs, the phosphorylation of H2AX (γH2AX) is located in the chromatin domains affected by chemical agents (Sirbu et al., 2011). In the normal mouse spermatocytes, γH2AX signals appear during the leptotene stage and accumulate on unsynapsed chromosomes by the zygotene phase, and then foci on the autosomal SCs gradually disappear during the pachytene stage, but globular γH2AX stains are still evident on the sex body (Xu et al., 2011).

In this study, besides that unsynapsed sex chromosomes were typically stained by γH2AX foci, we observed that γH2AX foci also presented on autosomal SCs in pachytene spermatocytes after HU treatment, indicating the accumulation of incomplete repair or unrepaired DSBs on autosomal SCs (Ayarza et al., 2016). In a clinical case, a meiotic DSB repair defect was reported in an azoospermic patient (Sciurano et al., 2006). Thus, such unrepaired DSB in meiosis may increase the risk of infertility. Moreover, unrepaired DSB also affected SC assembly in mice (de Vries et al., 2005). We found that the number of γH2AX foci significantly increased in mice treated with the high dose, suggesting that the high dose of HU treatment promoted the phosphorylation of H2AX and increased DNA damage. Notably, γ-rays and chemical agents were reported to cause additional γH2AX foci at the pachytene stage (Chicheportiche et al., 2007). Thus, DNA replication stress by HU could contribute to enhancing the formation of γH2AX foci (Ward and Chen, 2001; Arlt et al., 2012).

Furthermore, we analyzed the stage of γH2AX foci in the pachytene nuclei and found that both S-foci and L-foci in the early pachytene stage had a higher number of γH2AX foci than in the late pachytene stage in the HU-treated mice, compared with controls. These results confirmed a previous observation that greater numbers of S-foci per cell presented at early pachytene and L-foci were much less frequent at the late pachytene stage (Chicheportiche et al., 2007), suggesting that autosomal SCs with S-foci were more frequent when unrepaired DNA damage occurred. Additionally, these accumulations of DNA damage or chromosome fragility under replication stress by chemical agents frequently presented chromosomal abnormalities (Garcia-Cruz et al., 2009; Passerini et al., 2016; Yang et al., 2019).

Furthermore, the distribution of γH2AX foci in SCs was investigated. Our data further confirmed that γH2AX foci were distributed near the telomere region. In particular, a large number of γH2AX foci were stained in the sub-telomeric regions and distributed on the telomere-adjacent chromatin. This possible explanation is that HU-induced replication stress affected heterochromatin distribution and chromatin structure in the genome (Li et al., 2017). In mammalian cells, heterochromatin is crucial for male meiosis to maintain genome stability because the condensation structure of heterochromatin prevents DNA damage (Wu et al., 2009; Feng et al., 2016). Usually, γH2AX signals did not present in heterochromatic regions, but if DNA damage occurs in heterochromatin regions, it is difficult to be repaired due to the dense packaging of repetitive DNA sequences (Feng et al., 2017). DSB damage forming preferably in the periphery of telomeres and euchromatin was also reported (Kim et al., 2007; Klement and Goodarzi, 2017). The sub-telomeric and sub-centromeric regions near heterochromatin are prone to DSB damage, due to the alteration of heterochromatin structure (Snyder et al., 2009; Wang et al., 2016).

### Recombination Foci in Pachytene Spermatocytes of Mice Treated With HU

The mismatch repair protein, MLH1, is a marker of CO, which is required for the formation of meiotic COs and the evaluation of DSB repair in pachytene (Stern and Hotta, 1974; Guiraldelli et al., 2013). In wild-type pachytene spermatocytes of mice, at least one CO is presented on the chromosome arm (Jiang et al., 2018). To determine whether the formation of CO was affected by HU treatment, we evaluated the number of MLH1 foci in the pachytene of mice treated with HU. In control pachytene nuclei, the average number of MLH1 foci was 22.34 per cell on autosomal SCs in agreement with the published results (Anderson et al., 1999). However, in both the high- and low-dose groups, we observed that the number decreased to 17.74 and 17.62, respectively. In the clinical case, the analysis of MLH1 in infertile men demonstrated that non-obstructive azoospermia males have a significant reduction in the frequency of MLH1 foci compared with controls (Martin, 2008). A reduction in recombination foci in autosomal regions can lead to the abnormal disjunction of autosomal chromosomes, which in turn can cause a total or partial arrest of the meiotic process since deficiency in CO formation is difficult to correctly guarantee the chromosome segregation at metaphase I (Sarrate et al., 2014). In addition, SC length was considered as the best predictor of recombination rate, due to stable relative SC lengths throughout pachytene (Sebestova et al., 2016). We observed that the distributions of MLH1 foci for the long-length SCs were consistent with previous reports that there was a tendency toward a bimodal distribution pattern with peaks at the proximal and distal ends (Froenicke et al., 2002). For the short-length SCs, one MLH1 focus was distributed near the distal telomere, suggesting that one MLH1 focus may generate more frequently univalent chromosomes when the recombination process was affected (Sarrate et al., 2014).

In this study, a common feature of recombination foci in pachytene spermatocytes of mice treated with HU was demonstrated that a large proportion of MLH1 foci located near the distal ends of SC lengths, rarely close to centromeres, suggesting this distribution of MLH1 foci relied on the SC length (Anderson et al., 1999). Moreover, the distribution of MLH1 foci in infertile men was found near the telomere and sub-telomeric regions, suggesting these regions as the hot spots for the alteration of meiotic recombination (Ferguson et al., 2009). Overall, a reduction of recombination foci in male mice treated with the therapeutic dose of HU may affect the meiotic recombination event resulting in genome instability.

## Conclusion

In this study, the synapsis and DSB repair during the pachytene stage in mice spermatocytes after HU treatment were investigated. We observed that male mice treated with HU presented the increase of SC gaps and unrepaired DSB and also the reduction in the counts of recombination foci on the autosomal SCs in the pachytene spermatocytes. Moreover, unrepaired DSB and meiotic recombination sites were mainly distributed near the telomere region. These findings could provide some new insights into interpreting the effects of HU on spermatogenesis. Combined with previous studies, this study revealed that HU, an antineoplastic agent for the treatment of SCD, could have some effects on spermatogenesis which suggest possible problems in fertility. To better understand these abnormalities of the meiotic process, further analysis of testicular biopsy samples from patients with SCD undergoing HU therapy is suggested.

## Data Availability Statement

The original contributions presented in the study are included in the article/Supplementary Material, further inquiries can be directed to the corresponding author/s.

## Ethics Statement

The animal study was reviewed and approved by the Animal Care Committee at the University of Xuzhou.

## Author Contributions

XF and YZ designed this study and drafted the manuscript. BZ gave advice on HU administration. CZ searched the literature and investigated the studies. NW and YW contributed to review and editing of the manuscript. All authors reviewed the study findings and read and approved the final version before submission.

## Conflict of Interest

The authors declare that the research was conducted in the absence of any commercial or financial relationships that could be construed as a potential conflict of interest.

## Footnotes

1 https://imagej.nih.gov/ij/download.html

## Abbreviations

HU: hydroxyurea
SCD: sickle cell disease
DSBs: double-stranded breaks
SC: synaptonemal complex
CNV: copy number variants.
