# Supplementary material for: Therapeutic Dose of Hydroxyurea-Induced Synaptic Abnormalities on the Mouse Spermatocyte
Source: Front Physiol. 2021 Jul 9;12:666339. doi: 10.3389/fphys.2021.666339 (PMC8299468; doi:10.3389/fphys.2021.666339)
Supplement: Supplementary file 1 [file Data_Sheet_1.docx]

**Supporting Information**

**Table S1.** The average absolute and relative SC lengths were analyzed in the experimental and control groups; the mouse chromosome corresponds to the homologous human chromosome.

| SC index | Average absolute SC length with SD (µm) | | | Average relative SC length with SD (%) | | | Mouse Chromosome  index | Homologous human chromosome index |
| --- | --- | --- | --- | --- | --- | --- | --- | --- |
|  | 25 mg/kg | 50 mg/kg | Control | 25 mg/kg | 50 mg/kg | Control |  |  |
| 1 | 11.86 ± 3.32 | 12.99 ± 2.94 | 10.62 ± 1.41 | 8.26 ± 1.35 | 8.16 ± 1.04 | 7.64 ± 0.46 | 2 | 10/9/2/11/15/20 |
| 2 | 10.69 ± 2.60 | 11.75 ± 2.41 | 9.92 ± 1.20 | 7.45 ± 0.72 | 7.38 ± 0.62 | 7.14 ± 0.32 | 1 | 6/2/18/1 |
| 3 | 9.94 ± 2.31 | 10.99 ± 2.05 | 9.36 ± 1.11 | 6.92 ± 0.52 | 6.92 ± 0.49 | 6.74 ± 0.24 | 5 | 7/4/7/13 |
| 4 | 9.27 ± 2.01 | 10.38 ± 1.75 | 8.90 ± 1.04 | 6.47 ± 0.37 | 6.54 ± 0.29 | 6.41 ± 0.27 | 4 | 8/9/1 |
| 5 | 8.82 ± 1.91 | 9.93 ± 1.61 | 8.54 ± 1.00 | 6.15 ± 0.35 | 6.27 ± 0.25 | 6.14 ± 0.24 | 7 | 19/11/15/11/16/10/11 |
| 6 | 8.43 ± 1.70 | 9.45 ± 1.51 | 8.23 ± 0.89 | 5.89 ± 0.29 | 5.97 ± 0.25 | 5.93 ± 0.19 | 11 | 22/7/2/16/5/17 |
| 7 | 8.10 ± 1.55 | 9.10 ± 1.46 | 7.99 ± 0.89 | 5.67 ± 0.23 | 5.75 ± 0.27 | 5.75 ± 0.16 | 3 | 8/3/4/3/1/4 |
| 8 | 7.86 ± 1.49 | 8.75 ± 1.41 | 7.74 ± 0.82 | 5.50 ± 0.28 | 5.52 ± 0.26 | 5.58 ± 0.14 | 9 | 11/19/11/15/6/3 |
| 9 | 7.61 ± 1.40 | 8.45 ± 1.32 | 7.51 ± 0.77 | 5.33 ± 0.22 | 5.34 ± 0.24 | 5.41 ± 0.17 | 8 | 19/8/19/4/19/16/1 |
| 10 | 7.38 ± 1.34 | 8.20 ± 1.30 | 7.29 ± 0.75 | 5.17 ± 0.26 | 5.18 ± 0.21 | 5.25 ± 0.14 | 6 | 7/2/3/10/12 |
| 11 | 7.15 ± 1.29 | 7.95 ± 1.23 | 7.07 ± 0.75 | 5.01 ± 0.27 | 5.02 ± 0.22 | 5.09 ± 0.15 | 10 | 6/10/22/21/19/12 |
| 12 | 6.90 ± 1.25 | 7.68 ± 1.17 | 6.82 ± 0.78 | 4.84 ± 0.25 | 4.85 ± 0.21 | 4.91 ± 0.16 | 12 | 2/7/14 |
| 13 | 6.65 ± 1.23 | 7.37 ± 1.14 | 6.53 ± 0.72 | 4.66 ± 0.35 | 4.66 ± 0.24 | 4.70 ± 0.18 | 14 | 3/10/14/8/13 |
| 14 | 6.37 ± 1.16 | 7.07 ± 1.09 | 6.30 ± 0.72 | 4.47 ± 0.32 | 4.47 ± 0.25 | 4.53 ± 0.18 | 13 | 7/6/5 |
| 15 | 6.07 ± 1.10 | 6.73 ± 1.04 | 6.02 ± 0.65 | 4.26 ± 0.28 | 4.26 ± 0.25 | 4.33 ± 0.20 | 17 | 6/16/21/6/19/18/2 |
| 16 | 5.70 ± 1.05 | 6.33 ± 0.96 | 5.67 ± 0.65 | 4.01 ± 0.38 | 4.01 ± 0.31 | 4.09 ± 0.23 | 15 | 5/8/22/12 |
| 17 | 5.22 ± 0.87 | 5.79 ± 0.89 | 5.21 ± 0.57 | 3.68 ± 0.32 | 3.67 ± 0.32 | 3.76 ± 0.24 | 16 | 19/2/3/21 |
| 18 | 4.81 ±0.93 | 5.24 ± 0.83 | 4.85 ± 0.54 | 3.38 ± 0.38 | 3.32 ± 0.34 | 3.49 ± 0.22 | 18 | 10/18/5/18 |
| 19 | 4.07 ±0.84 | 4.29 ± 0.76 | 4.31 ± 0.56 | 2.86 ± 0.42 | 2.72 ± 0.33 | 3.10 ± 0.27 | 19 | 11/9/10 |


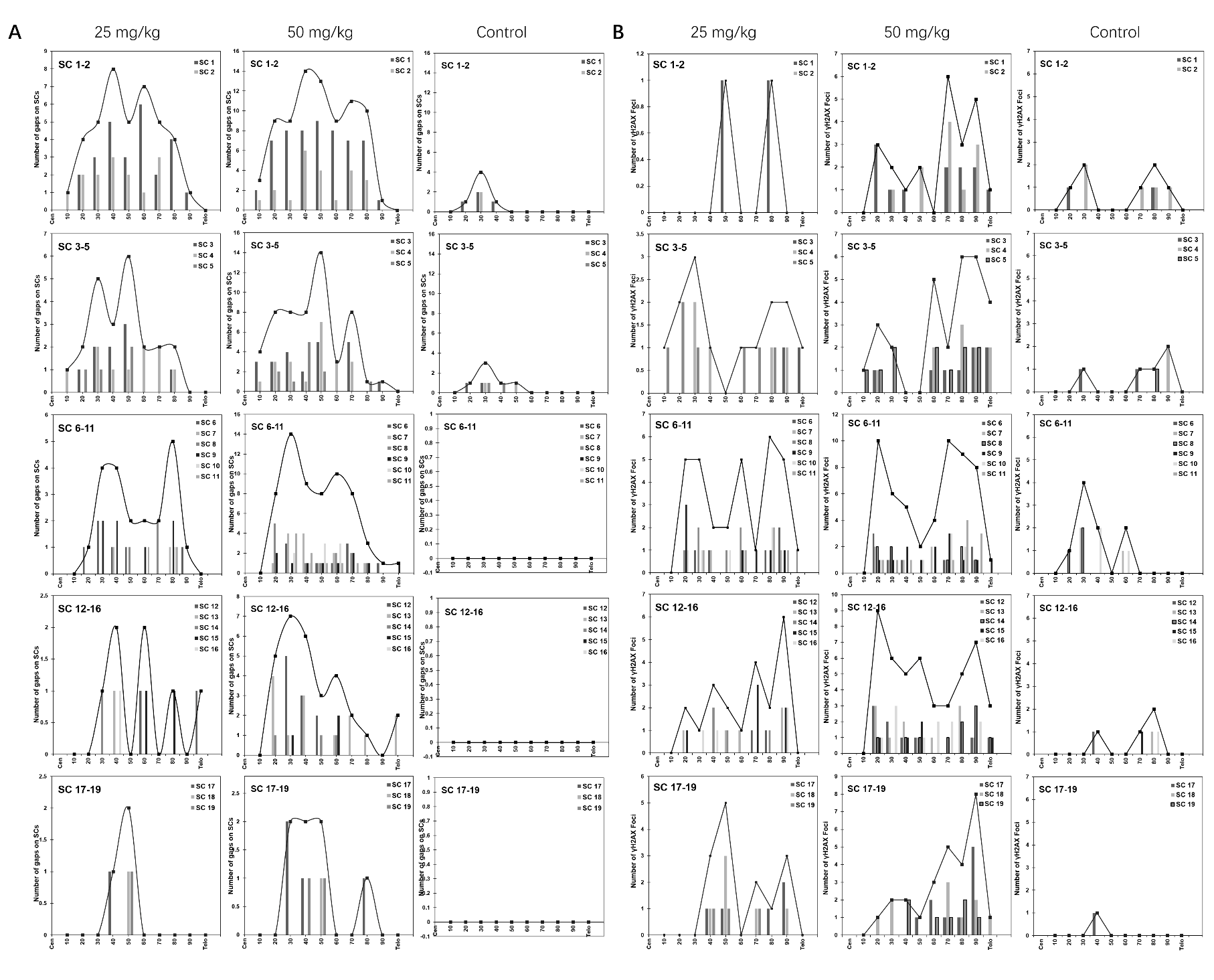


**Figure S1.** Distribution of gaps and γH2AX foci along with five SCs groups, according to the SC relative length (SCs 1 and 2, SCs 3–6, SCs 7–12, SCs 13–15, and SCs 16–19) from mouse spermatocytes (gaps: n = 100 SC sets; γH2AX foci: n=50 SC sets). For each graph, The X-axis represents the positions on the SCs from the centromeric end (left) to the telomere (right). The Y-axis indicates the number of gaps and γH2AX foci. (A) Distribution of gaps on autosomal SCs. The distribution of gaps on each SC is indicated by different color bars. The cumulative distribution of gaps in each group contains a smoothing curve above the histograms. (B) Distribution of γH2AX foci on autosomal SCs. The distribution of γH2AX foci on each SC is indicated by different color bars. The cumulative distribution of γH2AX foci on each group is indicated by the line above the histogram distributions.


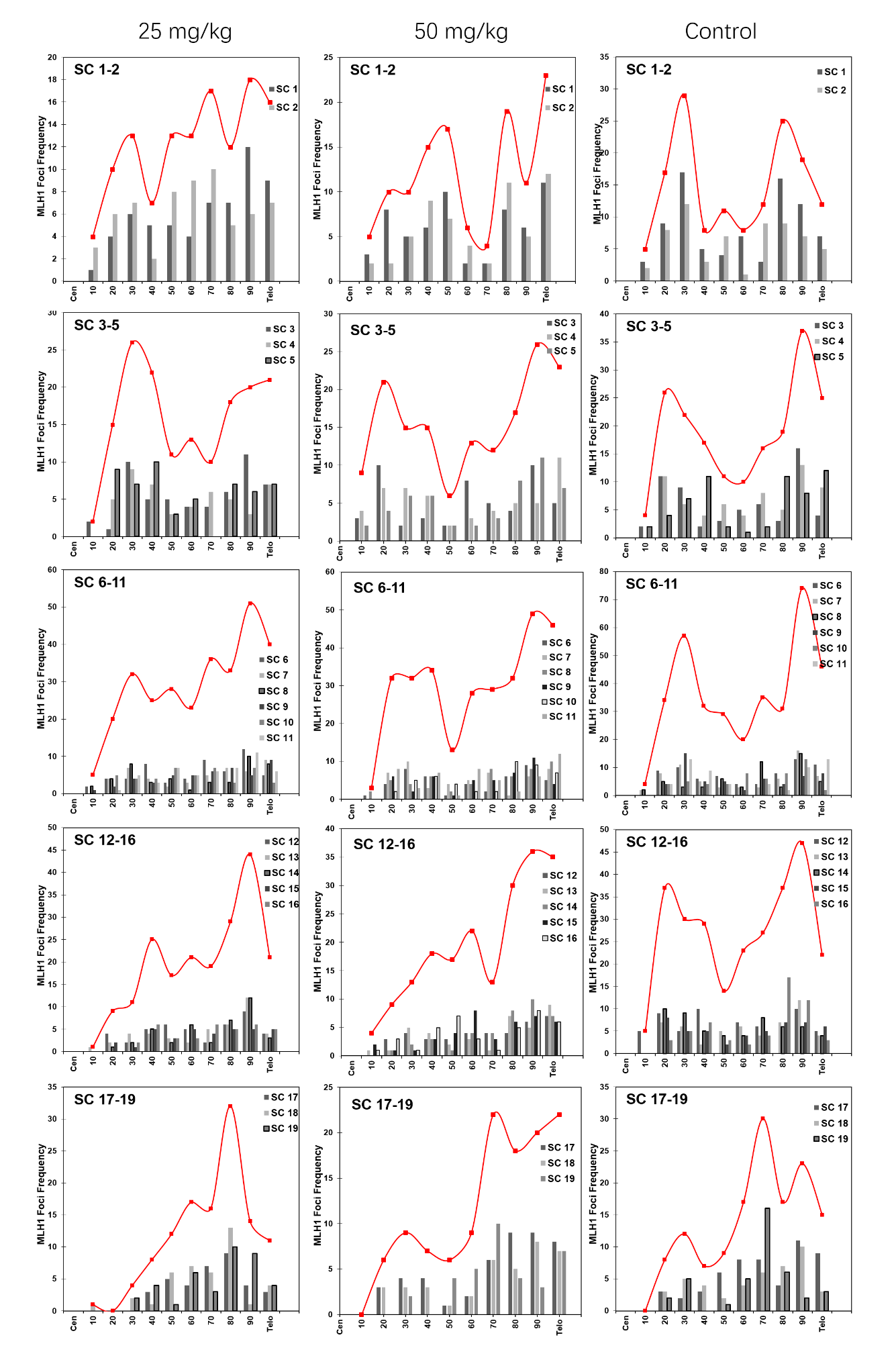


**Figure S2.** Distribution of MLH1 foci along with five SCs groups, according to the SC relative length (SCs 1 and 2, SCs 3–6, SCs 7–12, SCs 13–15, and SCs 16–19) from mouse spermatocytes (MLH1: n=50 SC sets). For each graph, The X-axis represents the positions on the SCs from the centromeric end (left) to the telomere (right). The Y-axis indicates the number of MLH1 foci. Distribution of MLH1 foci on autosomal SCs in 25mg/kg and 50 mg/kg HU and controls. The distribution of MLH1 foci on each SC is indicated by different color bars. The cumulative distribution of MLH1 foci on each group is indicated by the red line above the histogram distributions.

**
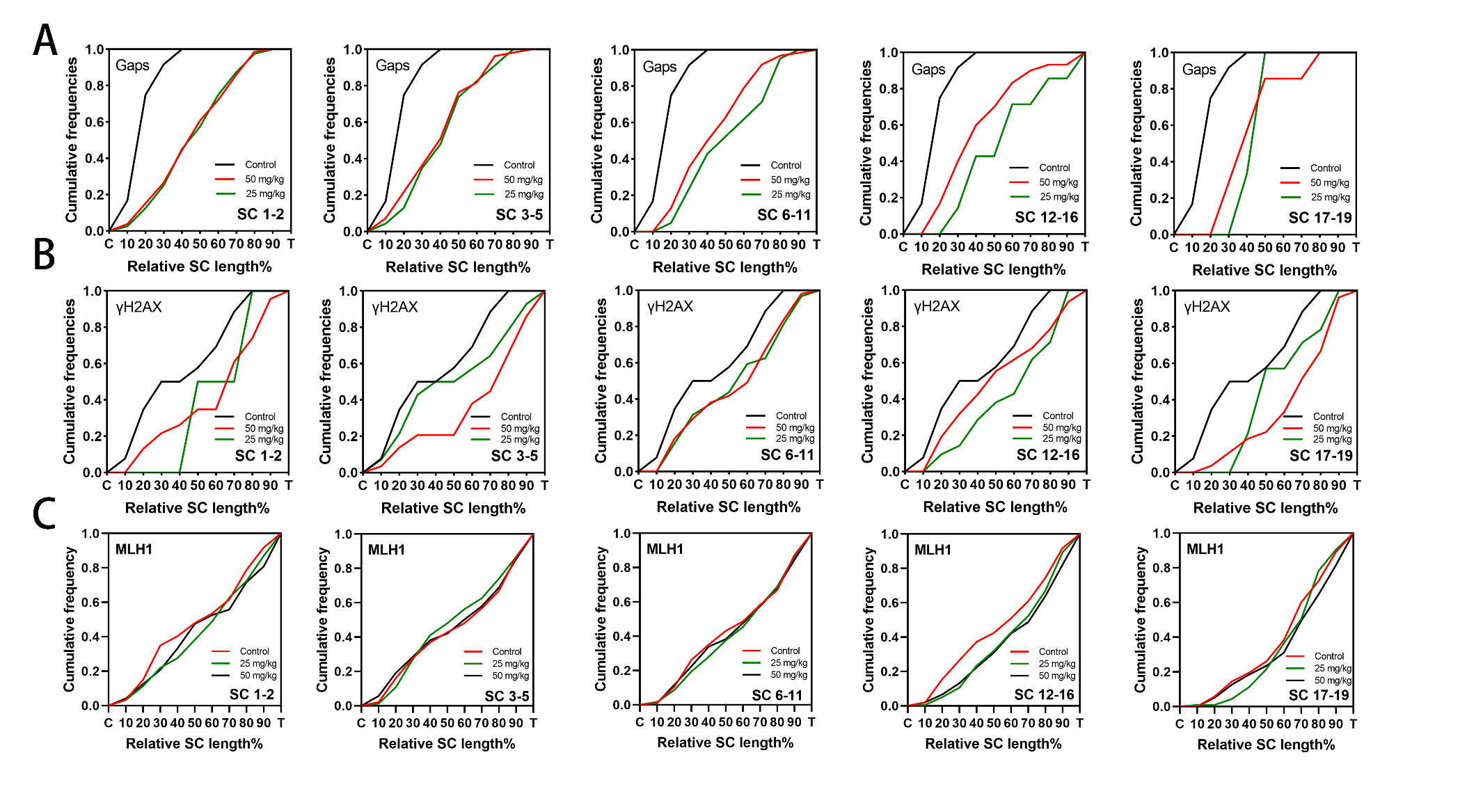
**

**Figure S3**. Distribution of gaps, γH2AX foci, and MLH1 foci along the SCs. Cumulative frequencies of gaps, γH2AX foci, and MLH1 foci are represented in A, B, and C, respectively. Distances to the centromere are expressed as percentages of the SC length. SC length index can be found on the lower right corner of each chart, besides the 25 mg/kg, 50 mg/kg, and control. For each SC group, there is no significant difference in the distribution of gaps, γH2AX foci, and MLH1 foci between experimental groups and control using a Kolmogorov-Smirnov test (n = 100; P>0.05).
